# Explanation of Increase in Prison Population

**Published:** 1911-07

**Authors:** 


### Explanation of Increase in Prison Population.

Accordingly, the marked increase in prison population, and especially in commitments, does not reflect an increase in crime but is largely accounted for by this difference in the scope of the two censuses.

The Census Bureau will be able later to segregate from the 1910 figures the cases of imprisonment for non-payment of fine and thereby obtain a figure which will be fairly comparable with the enumeration of six years ago. The larger number of admissions reported, as compared with the population present on January 1, is indicative of the fact that a large proportion of the commitments are for short sentences and for minor offenses. In the final census report the prisoners will be classified with reference to the offense for which sentenced and the term of sentence imposed.

The number of juvenile delinquents reported at the census of 1910 in institutions for that class was 22,903. This differs but little from the number reported in 1904, which was 23,034.

The number of paupers in alms houses on January 1, 1910, was 83,944. The number admitted during the year 1910 was 106,457, and the number discharged or dying during that year was 100,858. In 1904 the pauper population was 81,764 at the beginning of the year; the admissions during the year were 81,412; and the discharges or deaths, 77,886.

### STRIKING INCREASE OF INSANE POPULATION.

The enumeration of the insane in asylums indicates a very striking increase in this class of the population. In 1904 the number of insane in institutions was 150,151. In 1910 this number had increased to 184,123, an increase of 22.6 per cent in six years. The number of commitments to insane asylums during the year

1904 was 49,622, and during the year 1910 was 59,628, an increase of 20.2 per cent.

In 1904 the feeble-minded in institutions numbered 14,347; in 1910 the number was 20,199. The number of commitments to institutions for this class increased from 2599 in 1904 to 3848 in 1910.

